# Molecular Mechanisms of Plant Abiotic Stress Tolerance

**DOI:** 10.3390/ijms26062731

**Published:** 2025-03-18

**Authors:** Michael Moustakas

**Affiliations:** Department of Botany, Aristotle University of Thessaloniki, 54124 Thessaloniki, Greece; moustak@bio.auth.gr

## 1. Introduction

Global climate change compromises sufficient food production, and it is estimated that it may be reduced by 11–25% at the end of this century [1,2]. During their life cycle, plants are constantly exposed to numerous biotic and abiotic stresses that adversely influence plant growth and development, and also crop production [3,4]. Ensuring food security will demand mitigating or compensating the crop damages caused by exaggerating adverse environmental conditions [5,6,7,8].

Plants have developed several mechanisms that allow them to avoid and/or tolerate abiotic stresses through morphological and physiological adjustments [3,4,9]. The morphological adjustments that provide an escape from drought stress, for example, involve a decreased leaf area, an increased leaf thickness, leaf rolling or folding to minimize evapotranspiration, reduced stomatal number and conductance, and also an increased root system [9]. Drought physiological tolerance traits associated with the preservation of the water status involve osmotic adjustment through the accumulation of osmolytes that help plants acclimate to water deficit [9].

**Figure 1:**
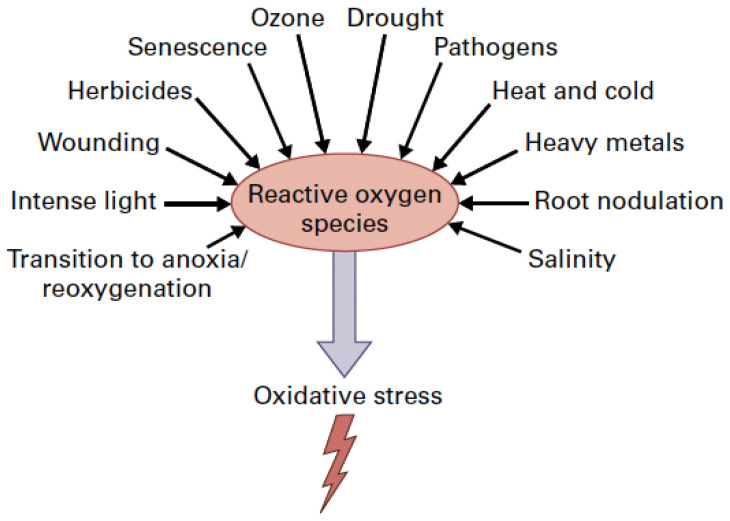
Environmental stress factors enhance the production of reactive oxygen species (ROS) that results in oxidative stress in plant cells (From [18] after license).

Environmental stress conditions result in oxidative stress induced by reactive oxygen species (ROS) accumulation (Figure 1) [10,11,12]. The superoxide anion radical (O_2_**^•^^−^**), the hydrogen peroxide (H_2_O_2_), and the singlet oxygen (^1^O_2_) are the main ROS produced in plant cells, mainly in the light reactions of photosynthesis, but are kept in homeostasis by the antioxidative enzymatic and non-enzymatic systems [12,13,14,15,16,17].

The beneficial effect of a small-dose or short-duration exposure to a stressor of an organism that is followed by a destructive effect at a larger dose or longer duration exposure to the same stressor has been termed “hormesis” [19,20,21]. However, a small-dose or short-duration exposure to a stressor that causes metabolic inhibition and a larger-dose or longer-duration exposure that results in metabolic stimulation have also been described as a hormetic response [22,23,24,25]. This Special Issue highlights some molecular mechanisms of plant tolerance to biotic and abiotic stresses that can assist in the development of realistic interventions for increasing agricultural productivity.

## 2. Mechanisms to Abiotic Stress Tolerance

As one of the most essential macro-nutrients for plant growth and development, nitrogen (N) is a key aspect that regulates crop yield [26], and nitrogen use efficiency (NUE) is important for sustainable agriculture [27]. Soto-Cerda et al. [27] evaluated 123 accessions of *Linum usitatissimum* L. (flax) at the seedling stage for NUE-related traits under optimum N and N deficiency. They explored the genetic architecture of NUE-related traits by means of a multi-omics approach integrating genome-wide association studies (GWASs), transcriptome analysis, and genomic selection (GS). QTL dataset identified the candidate genes of NUE-related traits for further studies and suggested genomic breeding tools to accomplish superior NUE under low N input in *Linum usitatissimum* L. [27]. The authors recommended the use of GWAS-derived QTL associated with a target trait since it is the most precise, cost-effective, and computationally advantageous for NUE and other quantitative traits in flax [27].

The main source of the important element N for terrestrial plants is nitrate (NO_3_−), which plants absorb from the soil using specific transport mechanisms in the plasma membranes of the root cells [28]. Under low nitrate concentrations in soils that limit plant growth, nitrogen supply is displayed by the high-affinity NO_3_− transporters of the NRT2 family [29]. By cloning two genes of the high-affinity NO_3_− transporters, *SaNRT2.1* and *SaNRT2.5*, from the euhalophyte *Suaeda altissima* to *Arabidopsis thaliana,* Khramov et al. [30] observed an increased expression of both genes under 500 mM NaCl (15-fold rise in SaNRT2.1 and 150-fold for SaNRT2.5). They suggested that SaNRT2.5 warrants a successful NO_3_− uptake by roots and functions as a fundamental high-affinity nitrate transporter under N deficiency of *S. altissima* plants [30]. A successful nitrate uptake and the consequential nitrogen assimilation serve as a feasible energy dissipation path safeguarding effective photosynthesis under high light conditions [31].

Jacalin-related lectins (JRLs) are broadly distributed in plants and are engaged in plant development and abiotic stress responses [32]. Quan et al. [32] examined the roles of JRLs in barley’s response to N deficiency and identified 32 *HvJRL* genes distributed at both ends of the seven barley chromosomes. Using transcriptome analysis, they identified in two barley genotypes with different N deficiency tolerance nine differentially expressed genes (DEGs) that encoded eight HvJRL proteins and suggested that the identified HvJRL DEGs could offer new candidate genes for tolerance to N deficiency [32].

Defoliation, which is the premature removal of leaves by cutting or grazing, is considered an unavoidable abiotic stress for forage and turf grasses [33]. Vegetative regrowth following defoliation is a decisive trait defining the persistence and productivity of these grasses [33]. Regrowth vigor is associated with photosynthetic ability, especially at the early stages of defoliation [33]. Sakashita et al. [33] using physiological and genetic analyses concluded that the regulation of vegetative regrowth upon defoliation in the newly emerged leaves is initiated by the de novo carbohydrate synthesis and the proper carbohydrate management.

Heat stress negatively impacts plant growth, development, and grain yield [34]. *Zea mays* (maize) production is severely affected by heat stress that is usually accompanied by drought stress [35]. The transcription factor ZmNF-YA1, which was identified as a positive regulator of the drought stress response in maize [36], was also identified to contribute to maize thermotolerance [35].

Cold stress is among the harmful abiotic stresses that affect plant growth and development, limiting the geographical distribution of plants and reducing plant quality and productivity [37]. Dou et al. [38], using RNA sequencing, identified 24,695 differentially expressed genes (DEGs) in two cultivars of a tropical ornamental flower (*Anthurium andraeanum*) that had contrasting cold tolerance. From these DEGs, 9132 were common in the tolerant and susceptible cultivar, providing a basis for elucidating the mechanism of cold tolerance in *A. andraeanum* and the potential targets for molecular breeding.

## 3. Biostimulants in Plant Abiotic Stress Tolerance

The agricultural industry has increasingly been focusing on the utilization of biostimulants for enhancing plant growth and crop production under both non-stress and stress conditions [39,40,41]. Owing to global climate change, agricultural yields have been significantly reduced, affecting global food security [42]. Melatonin (MT) molecules are considered to be hormones having a widespread distribution from prokaryotic bacteria to higher plants, which can be used to enhance crop yield by acting as photosynthetic biostimulants [41,43]. Muhammad et al. [44], reviewing the current literature on the beneficial impact of MT on plants under abiotic stress conditions, reported that under drought stress conditions, MT improved water use efficiency and reduced transpiration rate. They also described that MT employs protective effects against low humidity, heavy metal stress, nutrient deficiency, salinity, extreme temperatures, and waterlogging by upregulating the photosynthetic function and balancing ROS production [44].

Wu et al. [45], in a review article, highlight the pivotal role of MT and mepiquate chloride (MC) in regulating cotton growth and yield under abiotic stress conditions, emphasizing specifically cotton’s morpho-physiological and biochemical activities and their biosynthetic signaling and transduction pathways. Cotton (*Gossypium hirsutum* L.), cultivated for the textile industry, is an economic crop that accounts for 35% of the worldwide fiber consumption [46]. MT and MC, by improving net photosynthetic activity, cell enlargement, antioxidant enzymes, and cytokinins, alleviated the adverse effects of abiotic stress in cotton and increased its yield [45].

Aspirin, which is extensively used in human health, can also be beneficial for plant health [47]. Aspirin is the trade name for acetylsalicylic acid, which, by hydrolysis, produces salicylic acid. Salicylic acid is a plant hormone produced in plant chloroplasts and its concentration is enhanced in response to biotic or abiotic stresses [48]. Aspirin foliar spray in tomatoes acts osmoregulatory, offering antioxidant protection, keeping a higher fraction of open reaction centers, and enhancing photosystem II (PSII) photochemistry (Φ*_PSII_*) [47]. Furthermore, it reduces ROS formation, decreasing the excess excitation energy on PSII and by acting as a photosynthetic biostimulant it can enhance crop yield [47].

## 4. Abiotic Stress Signal Recognition and Transduction

**Figure 2:**
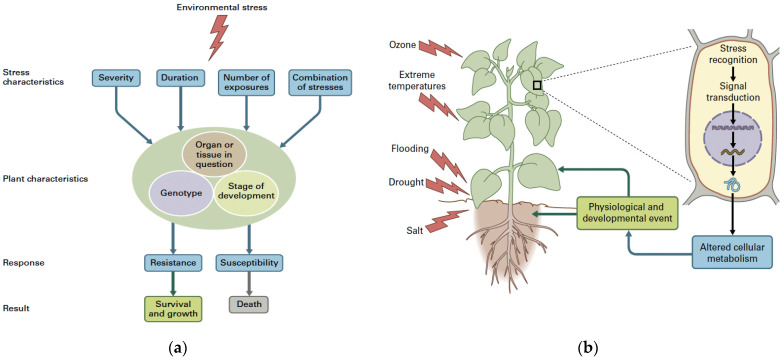
The response of plants to an environmental stress factor depends not only on the severity, the duration, the rate (number of times) at which the stress is imposed, and on the synergistic or antagonistic impact of stress combination, but also on the plant species, the organ or tissue affected, the developmental stage, and the genotype in question. The response to the stress can result in acclimation or susceptibility. Failure to counteract severe stress can result in plant death (From [18] after license) (**a**); the different environmental stress signals are recognized from the plant and the signals are transferred within cells and through the plant. After environmental stress recognition, the signals usually result in modified gene expression, which in turn impacts plant metabolism and development at the whole plant level (from [18] after license) (**b**).

The response of plants to abiotic stress factors depends not only on the severity, the duration, the rate (number of times) at which the stress is imposed, and on the synergistic or antagonistic impact of stress combination, but also on the plant species, the organ or tissue affected, the developmental stage, and the genotype in question [49]. Failure to counteract a severe stress can result in the plant death (Figure 2a). Environmental stress signals that are received and recognized by plants are communicated throughout the plant and within cells (Figure 2b). After environmental stress recognition, the transduction of signals results in altered gene expression, which in turn modifies metabolism and plant development [49]. Global climate change severely impacts the information perceived by plants affecting plant physiology, defense, and development [4].

To perceive stress signals and to transduce these signals to intracellular responses, plants rely on protein kinases that initiate protein phosphorylation reactions which lead to structural changes in proteins, modulating their activity [50,51]. Protein kinases, like mitogen-activated protein kinases (MAPKs), calcium-dependent protein kinases, or receptor-like kinases (RLKs), play a fundamental role in modulating plant growth and development during abiotic stress [52,53]. Gandhi and Oelmüller [53], in a review article, discuss the available information on membrane-bound receptor-like kinases (RLKs), their role in abiotic stress tolerance, and their role in influencing plant–environment interactions, and they suggest possible novel approaches to engineer stress-tolerant crop varieties [53].

Plants recognize light signals through photoreceptors, between which phytochromes are the primary receptors responsible for absorbing red light (R)/far-red light (FR) [54,55]. Phytochromes are not only involved in regulating plant growth and development but also they play a vital role in facilitating plants to handle different abiotic stresses such as drought, salinity, high/low temperatures, and high/low light intensity [56]. Qiu et al. [56] reviewed recent studies on the mechanisms of action of phytochromes in plant stress tolerance and discussed the importance of modulating the genes involved in the phytochrome signaling pathways to coordinate plant growth, development, and stress responses.

The class of the peptide hormones C-TERMINALLY ENCODED PEPTIDEs (CEPs) play a principal role in regulating the response of plants to abiotic stress [57,58]. Mei et al. [59] identified 54, 59, 34, and 35 CEP genes from *Gossypium hirsutum* (2n = 4x = 52, AD1), *G. barbadense* (AD2), *G. arboreum* (2n = 2X = 26, A2), and *G. raimondii* (2n = 2X = 26, D5), respectively, and categorized cotton CEP genes into two subgroups based on their domain differentiation. They concluded that cotton CEP genes are generally expressed throughout the whole plant, but some genes display specific expression patterns [59].

Song et al. [60] examined the characteristics and functional divergence of universal stress proteins (USPs) in blueberry (*Vaccinium corymbosum*). Universal stress proteins (USPs) perform important roles in hormonal regulation and plant development, and also in abiotic stress responses [60]. Song et al. [60] identified 72 *VcUSP* genes from the Genome Database of Vaccinium that could be divided into five groups based on their phylogenetic relationships [60]. From the 72 *VcUSP* genes, 21 *VcUSPs* responded to UV-B radiation, 7 responded to exogenous ABA, and 2 could act as bridges integrating UV-B and ABA signaling [60].

The cuticle that covers the outer epidermal surface of most above-ground tissues of plants prevents water loss, forming a diffusion barrier that limits water and solute transport, and protects the plants against pest and pathogen attack, and from chemical and mechanical damage, contributing to environmental adaptation [61,62]. However, even when stomata are completely closed, leaves still lose water, which can be defined by the leaf minimum conductance [63]. The cuticular wax biosynthesis was revealed to be regulated at the transcriptional level [64]. Liu et al. [65] identified the wheat MYB transcription factor TaMYB30 as a transcriptional activator that positively regulates wheat wax biosynthesis, probably via the transcriptional activation of TaKCS1 and TaECR. Increasing photosynthetic photon flux density (PPFD) was reported to increase cuticular wax deposition without altering the relative lipid composition or the rate of foliar water loss [66]. However, cuticular thickness, which was found to vary inversely with high temperatures, may impact photosynthetic capacity [67].

Karst regions, portrayed by a thin soil layer, are sensitive to climate change effects, especially changes in precipitation which can affect the survival of most at-risk species, leading to the extinction of particularly small plant populations [68,69]. Wu et al. [70] explored the molecular mechanisms involved in the drought tolerance of the endemic in the karst mountain areas, medicinal species *Illicium difengpi* (Schisandraceae), and concluded that abscisic acid (ABA), methyl jasmonate (MeJA), and zeatin riboside (ZR) play a regulatory role in the drought tolerance response of *I. difengpi* plants, while they identified by transcriptomics the key genes involved in the signal transduction [70].

## 5. Crop Damage by Biotic Stress

Biotic stress is estimated to reduce universal crop production by 5–30%, and the damage can be as high as 50% in the lack of insecticide application [71]. Aphids, one of the most noteworthy pests in worldwide agriculture, through sucking phloem sap, cause damage to crops and other plants, spreading plant viruses, and reducing photosynthetic efficiency [72]. Cytochrome P450 monooxygenases (*CYP450s*) accomplish a range of physiological roles, including pesticide resistance [73]. Wang et al. [72] identified a total of 1100 *CYP450* genes at the whole genome level in 19 aphid species. Differential expression analysis of *CYP450* genes in the cereal crop aphids *Sitobion miscanthi*, *Schizaphis graminum*, *Rhopalosiphum padi*, and *Diuraphis noxia* provided evidence of organ specificity genes, tissue specificity genes, growth stage specificity genes, and detoxification metabolic genes among the four cereal crop aphids [72].

## 6. Conclusions

This Special Issue comprises 17 articles covering the different molecular mechanisms of abiotic stress tolerance. However, due to global climate change, which exaggerates the adverse environmental conditions, the plant tolerance mechanisms to abiotic stresses need additional investigation for a better understanding of the responses of plants to stress factors. This can help in the development of realistic interventions for increasing agricultural productivity.
